# Effects of competitive physical activity on serum irisin levels and bone turnover markers

**DOI:** 10.1007/s40618-021-01529-0

**Published:** 2021-03-06

**Authors:** A. Gaudio, R. Rapisarda, A. Xourafa, L. Zanoli, V. Manfrè, A. Catalano, S. S. Signorelli, P. Castellino

**Affiliations:** 1 Department of Clinical and Experimental Medicine, University of Catania, AOU Policlinico “G. Rodolico - San Marco”, Via S. Sofia 78, 95123 Catania, Italy; 2 AOU Policlinico “G. Rodolico - San Marco”, Catania, Italy; 3 Clinica del Mediterraneo, Ragusa, Italy; 4 Department of Clinical and Experimental Medicine, University of Messina, Messina, Italy

**Keywords:** Muscle, Bone, Myokines, Irisin, Physical activity, Football

## Abstract

**Background:**

Irisin, a myokine, is a polypeptide derived from the cleavage of the extracellular domain of fibronectin domain-containing protein 5, a receptor that is present on different tissues (skeletal muscle, pericardium, myocardium, and brain), whose functions are not yet fully defined.

**Purpose:**

The main aim of our study was to evaluate the effect of competitive physical activity on serum irisin levels and bone turnover markers.

**Methods:**

Fifteen male footballers and an equal number of subjects of the same age and gender, but with a predominantly sedentary lifestyle, had their serum levels of irisin and bone turnover markers measured. Bone mineral status was evaluated in both groups by quantitative bone ultrasound of the calcaneus. In addition, only in footballers, biochemical analyses were repeated after 3 months.

**Results:**

We did not observe significant differences in the serum levels of calcium, phosphorus, and parathyroid hormone between the two groups. The footballers had significantly higher quantitative bone ultrasound, 25-OH vitamin D, and creatinine values than the controls. There were also no significant differences in the bone alkaline phosphatase, carboxy-terminal telopeptide of type I collagen, osteoprotegerin, sclerostin or Dkk-1 values, while the irisin levels (+ 89%, *p* < 0.001) and RANKL were significantly higher in the footballers compared to those in the controls.

**Conclusion:**

Our study shows that footballers have significantly higher serum irisin values than the general population. Irisin could be the "trait d’union" between bone health and physical activity.

## Introduction

For years, musculoskeletal interaction has only been considered in terms of its mechanical aspect. In fact, the discovery that bone and muscle cells communicate on both biochemical and molecular levels, and not just mechanically [1], has led to new insights. Bone and muscle were considered to be two organs that only allowed locomotion and movement. In recent times, numerous studies have shown that they are also metabolic regulators; in particular, skeletal muscle tissue is a reserve of amino acids and glucose [2, 3], while bone tissue stores ions for metabolic processes [4, 5]. Thus, scientists have shown that muscle and bone cells communicate with each other biochemically and molecularly [1]. This close relationship can already be seen during embryonic development—both tissues are formed from somites of the paraxial mesoderm, and they share organogenesis, during embryonic development, through a closely related gene network [6]. Recently, the concept of the "bone-muscle unit" [7, 8] was introduced due to the close correlation between bone geometry and muscle mass; consequently, an increase in muscle strength always precedes an increase in bone strength. The bone acts through the production of sclerostin and osteocalcin, while muscle produces various factors, such as myostatin, IL-6, and irisin, which are called myokines and act on various organs [9, 10]. It is widely recognized that exercise has beneficial consequences on certain diseases, such as diabetes, obesity, cardiovascular disease and osteoporosis [11]. During exercise, muscle secretes myokines, which affect various organs (including bone tissue) [9–12]. One widely studied myokine, in particular, is irisin, whose name has been proposed by Bostrom et al. [13], from the goddess of Olympus, Iris or Iride, the messenger of the gods, to indicate the presumed role of messenger of the endocrine signal. Irisin concentration is inversely proportional to fragility fractures in postmenopausal women [14], and there is an inverse correlation with sclerostin concentration, regardless of age and gender [15]. Furthermore, during physical activity, an increase in irisin concentration has been shown, especially after anaerobic activity compared to aerobic activity [16], with an increase in bone density and strength in athletes [17].

Previous research has indicated that football training is associated with a positive impact on bone mineral density (BMD) evaluated by dual X-ray absorptiometry (DXA) [18, 19]. Albeit DXA is considered the gold standard tool in assessing osteoporosis, evidence supports the use of quantitative ultrasound (QUS) in evaluating bone status. In particular, QUS of the calcaneus is a radiation-free, low-cost, rapid, and easily usable technique for the measurement of bone parameters that are highly correlated with DXA [20].

The main aim of our study was to evaluate the effects of competitive physical activity on serum levels of irisin and bone turnover markers in a group of footballers and compare them with a control population of the same age and gender, who were not dedicated to sports. We also studied the modifications of these parameters in footballers during the sporting season. A secondary aim of the study was to evaluate the effects of physical activity on bone mineral status using the QUS technique.

## Materials and methods

### Subjects

We enrolled 15 male footballers belonging to the S.S.D. Acireale Calcio 1946 S.R.L. The exclusion criteria were: (1) use of drugs that affect bone metabolism, including bisphosphonates, in the last 12 months; (2) chronic use of glucocorticoids for more than 3 months; (3) known bone diseases (Paget's disease, rheumatoid arthritis, hyperparathyroidism, hypercortisolism, malignant tumours, renal bone disease, chronic liver disease, and post-transplant bone disease); (4) history of diabetes or serious cardiovascular disease (myocardial infarction or uncontrolled hypertension); or (5) any bone fracture within the past 2 years. In addition, a control group of 15 subjects of the same age and sex was recruited, with a predominantly sedentary lifestyle. The same exclusion criteria indicated for the footballers were used for the controls.

The beginning of the study was communicated to the local ethics committee, which was noted in the meeting of 23 July 2010. Prior to participating in the study, all subjects provided informed consent.

### Laboratory analysis

At the time of enrolment, before the beginning of the local championship, all subjects provided a blood sample, which was taken in the morning after a night of fasting. In addition, a repeat blood sample was taken for the footballers, 3 months later (during the championship).

Laboratory blood tests were performed either in the two hours following the collection of blood samples or on thawed serum, which had been stored at − 30 °C. Serum concentrations of total calcium (corrected for albumin concentration), phosphorus and creatinine were measured using standard automated laboratory techniques. Serum levels of 25-OH vitamin D, parathyroid hormone (PTH), bone alkaline phosphatase (B-ALP), type I collagen carboxy-terminal telopeptide (CTX), osteoprotegerin (OPG), RANKL, Dkk-1, sclerostin and irisin were dosed. 25-OH vitamin D was assayed by enzyme immunoassay (IDS Ltd., UK), and the intra- and inter-assay precision were < 8% and < 10%, respectively. Serum PTH levels were measured by enzyme immunoassay (BioSource Europe S.A., Belgium), and the intra- and inter-assay precisions were 1.1% and 7.1%, respectively. B-ALP and CTX were measured using the immune enzymatic kits of Immunodiagnostica System Ltd (Fountain Hills, AZ). The intra- and inter-assay precisions were between 1.8 and 10.8% for both tests. Serum OPG levels were measured by enzyme immunoassay (Biomedica Gruppe, Austria), and the intra- and inter-assay precision were 4% and 8%, respectively. Serum levels of total RANKL were determined by enzyme immunoassay (Biovendor, Czech Republic), and the intra- and inter-assay precision were 10.5% and 12.5%, respectively. Dkk-1 was measured with an ELISA method using reagents supplied by Biomedical (Vienna, Austria); the intra-assay precision was 7–8% and the inter-assay precision was 9–12%. Sclerostin was measured by an ELISA method using Biomedical reagents (Wien, Austria); the detection limit of the method was 0.18 ng/mL, and the intra-assay CV was between 6 and 10%. Irisin was dosed using the Elisa kit supplied by Biovendor (Czech Republic); the detection limit of the method was 1 ng/mL and the intra- and inter-assay precisions were < 8% and < 10%, respectively.

### QUS of the calcaneus

Bone mineral status was assessed in all subjects by the right heel QUS method using the Achilles Express ultrasound device (GE Lunar, Madison, WI). This provides three ultrasound bone parameters: attenuation (broadband ultrasound attenuation, BUA), speed of sound (SOS), and stiffness (SI). SI derives from the combination of BUA and SOS, based on the following SI algorithm = 0.67 × BUA + 0.28 × SOS – 240.

A quality control procedure was performed daily, before the measurements. In vivo, the short-term precision on 10 healthy subjects, calculated on three measurements repeated by the same operator, with repositioning, and expressed as the average square root of the variation coefficients, was 2.05% for SI. A single ultrasound device was used during the study, and all measurements were performed by the same specialized technician (AG).

### Statistical analysis

Descriptive statistics and significance levels were analysed using Analyze-it standard edition 5.65.7. The data for continuous variables are expressed as means ± SD. The normal distribution of values was verified for the different parameters with the Kolmogorov–Smirnov test. Pearson linear regression analysis (normal distribution) or the Spearman test (non-normal distribution) were used for association studies. Comparisons between continuous variables between groups were made using the Student's *t* or Wilcoxon test. The comparison of categorical variables between groups was performed using the *χ*^2^ test. A *p* value of 0.05 was considered statistically significant.

## Results

**Fig. 1:**
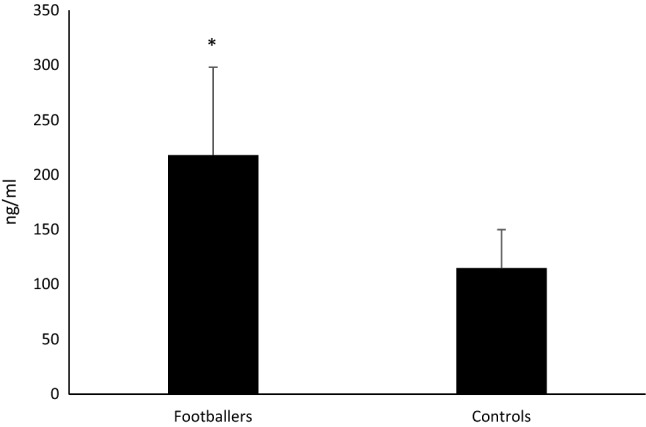
Irisin levels in footballers and controls at baseline. *p < 0.001

The clinical characteristics of footballers and controls are presented in Table 1. There were no significant differences in age, anthropometric characteristics and smoking habits between the two groups. The biochemical and ultrasound data of the study population are depicted in Table 2. There were no significant differences between the two groups with regard to calcium, phosphorus and PTH. However, footballers had significantly higher values of 25-OH vitamin D and creatinine than the controls. There were also no significant differences in sclerostin, Dkk-1, OPG, B-ALP and CTX values, while RANKL and irisin levels (Fig. 1) were significantly higher in footballers compared to those in the controls.

**Table 1:** Study population characteristics

	Footballers	Controls	*p*
*n*	15	15	–
Age	22.6 ± 4.2	22.0 ± 3.6	ns
BMI (Kg/m^2^)	22.87 ± 0.84	23.54 ± 1.84	ns
Smoking	1	2	–

Data are expressed as mean ± SD (where possible)

**Table 2:** Biochemical and ultrasound data of footballers and controls

	Footballers	Controls	*p*
Calcium (mmol/l)	2.51 ± 0.08	2.46 ± 0.09	ns
Phosphorus (mmol/l)	1.10 ± 0.14	1.12 ± 0.15	ns
PTH (pg/ml)	38.42 ± 17.76	31.26 ± 8.78	ns
25 OH Vitamin D (ng/ml)	36.23 ± 15.88	23.78 ± 7.78	< 0.05
Creatinine (mg/dl)	1.01 ± 0.07	0.78 ± 0.12	< 0.05
Sclerostin (pmol/l)	52.2 ± 9.9	49.9 ± 11.1	ns
Irisin (ng/ml)	218 ± 80	115 ± 35	< 0.001
Dkk-1 (pmol/l)	8.06 ± 1.87	7.38 ± 1.24	ns
OPG (pmol/l)	1.86 ± 0.41	1.95 ± 0.75	ns
RANKL (pmol/l)	1.002 ± 0.693	0.512 ± 0.393	< 0.05
B-ALP (ng/ml)	14.47 ± 6.64	13.75 ± 7.33	ns
CTX (ng/ml)	0.697 ± 0.127	0.742 ± 0.525	ns
Stiffness (%)	96.3 ± 4.1	93.0 ± 3.5	< 0.05

Data are expressed as mean ± SD

Irisin levels were correlated neither with age nor with BMI in any group. As far as bone mineral status is concerned, footballers had significantly higher SI values than the controls (Table 2).

In footballers, after 3 months of training (Table 3), a significant reduction in sclerostin values was observed, together with an increase in B-ALP, while irisin levels and other assessed parameters did not change significantly.

**Table 3:** Biochemical parameters in footballers after 3 months of training

	Baseline	After 3 months of training	*p*
Sclerostin(pmol/l)	52.2 ± 9.9	45.6 ± 8.9	< 0.05
Irisin (ng/ml)	218 ± 80	225 ± 72	ns
Dkk-1 (pmol/l)	8.06 ± 1.87	8.21 ± 3.70	ns
OPG (pmol/l)	1.86 ± 0.41	1.87 ± 0.95	ns
RANKL (pmol/l)	1.002 ± 0.693	0.986 ± 0.591	ns
B-ALP (ng/ml)	14.47 ± 6.64	22.48 ± 12.81	< 0.05
CTX (ng/ml)	0.697 ± 0.127	0.711 ± 0.313	ns

Data are expressed as mean ± SD

## Discussion

Our study shows that footballers had significantly higher irisin values (+ 89%, *p* < 0.001) compared to the controls. Given the similar age and anthropometric characteristics between the two groups, it can be reasoned that this increase is due to the more marked physical activity carried out by the footballers. This is in agreement with Kurdiova et al. [21], who demonstrated that irisin levels are related to the degree of physical activity usually performed and to muscular strength, contractility and volume. During the 3 months of training, the serum levels of irisin in footballers did not change significantly. This only partially agrees with a study performed by Anastasilakis et al. [22], which demonstrated that basal levels of irisin are not related to the degree of physical activity, but increase after 20 min of intense muscle exercise. According to Loffler et al. [23], irisin levels increase after acute and strenuous exercise, but they do not change after long-term exercise (6 weeks/1 year). Physical activity induces the activation of the peroxisome proliferator-activated receptor-gamma coactivator-1α (PGC-1α) [7, 24], which determines the synthesis of fibronectin domain-containing protein 5 (FNDC5). FNDC5 is a receptor, whose functions are not yet fully defined. It is present on several tissues, including skeletal muscle, pericardium, myocardium and brain, and seems to be of critical importance for the differentiation of myoblasts and neurons [24–26]. It consists of 209 amino acid residues, where an N-terminal sequence and a C-terminal intracytoplasmic segment consisting of 39 amino acid residues can be distinguished [24]. Irisin is derived from the extracellular N-terminal portion that is released into the circulation during the proteolytic cleavage process [13, 24]. In recent years, literature has focused on the responses of irisin to various exercise patterns. Sprint-type exercises led to an acute increase in the peripheral concentration of irisin in dogs [27] and in humans [28, 29]. This could be due to a compensatory mechanism of the organism in the state of metabolic need, due to the reduction of muscle ATP during an acute effort [30]. Even high-volume resistance exercises on the whole body led to an increase in the concentration of irisin 1 h after exercise [16, 28, 31], while it remained unchanged when the exercise was performed on a single muscle district [32]. Furthermore, an increase in the concentration of irisin has been shown after vibration exercise [33].

A meta-analysis by Qiu et al. [34], evaluating three randomized controlled trials, showed that chronic resistance exercise training determines a moderate and significant effect in decreasing circulating irisin compared with the control, while endurance exercise training has only a similar but not significant trend. The authors [34] also observed, in nine non-randomized studies, that chronic exercise training was associated with a small and non-significant overall effect in decreasing circulating irisin compared with baseline. In our study, irisin serum levels did not change in footballers during the championship. Since we did not evaluate the trend of this parameter in controls, we could not interpret this data comprehensively.

Several studies have reported that circulating irisin is positively associated with insulin resistance and fasting blood glucose [35, 36] in non-diabetic subjects, indicating that irisin might be involved in the regulation of glucose metabolism. All our study subjects were non-diabetic, but we did not have information on their food consumption or insulin sensitivity.

Our athletes had higher values of creatinine and 25-OH vitamin D than the controls. The first was probably due to the greater muscle mass of the footballers, and the second was probably a result of greater exposure to sunlight. The ultrasound examination of the calcaneus showed better values in the players compared to those in the controls, as previously observed by other authors and by Falk et al. [37] in particular, who showed that tibial SOS was higher in soccer players compared with that in controls. This confirms the beneficial effects of physical activity on bone and the possible effects of irisin on the bone. Indeed, Colaianni et al. [38] were among the first to prove it. They highlighted the ability of irisin to increase the differentiation of bone marrow stromal cells into mature osteoblasts. In particular, mice subjected to 3 weeks of exercise activity had a greater expression of irisin/FNDC5 in skeletal muscle tissue, which is a direct effect on the differentiation of osteoblasts in vitro and a greater expression of alkaline phosphatase and collagen I [38]. The same group subsequently demonstrated the direct action of irisin on bone in vivo. They showed that the tibias of young male mice treated with low doses of recombined irisin (r-irisin), which has no effect on fat, showed a 7.15% increase in cortical BMD compared with that in controls, improving the geometric bone architecture without modifying the trabecular bone [39]. All this is in line with previous literature, which showed a greater sensitivity of cortical bone to the action of physical activity compared to that of trabecular bone [40, 41]. In the same study, Colaianni et al. [39] also noted a reduction in sclerostin (a specific glycoprotein responsible for bone remodelling by inhibiting its formation [42]), assuming a direct load-mimetic action of irisin on osteocytes. Indeed, the concentration of irisin is inversely proportional to the concentration of sclerostin, regardless of age and gender [15], and a positive association between irisin and bone state has been demonstrated in healthy children [43]. This was confirmed by our study in which a decrease in sclerostin levels was observed after 3 months of training. At baseline, before the start of the competitive season, the bone turnover markers, as well as the inhibitors of the Wnt/beta-catenin signal (sclerostin and Dkk-1), appeared to be overlapping in footballers and controls, with the exception of the marked increase in RANKL, although this did not result in a stimulus for bone resorption. This increase is explained in a paper by Xiong et al. [44], where it was highlighted that the major source of RANKL is not osteoblasts as previously believed, but osteocytes. Therefore, we believe that the athlete, even if he has not recently completed a training cycle, has a basal osteocytic stimulation that is expressed as an increase in RANKL, an element that could favour the activation of bone turnover and be preparatory for the increase in new bone formation. In fact, after 3 months of training and competitive activity, a decrease in sclerostin levels is observed in footballers, as well as a marked increase in bone neoformation, as evidenced by the elevation of B-ALP.

Moreover, Colaianni et al. [45] observed a correlation between irisin and total or bone sub-regional BMD in soccer players. We did not show a correlation between irisin and QUS parameters, but this could be related to the different instruments (ultrasound vs. densitometric) and site of measurement (heel vs. whole body).

Finally, regarding bone mineral status, our footballers presented with significantly higher QUS values than the controls. This is in line with other studies that used the same technique and site of measurement [46]. This better ultrasound transmission on bone tissue of footballers may depend on physical properties of bone not measured by DXA but able to modulate bone strength, and this makes the QUS technique a useful tool in different clinical settings, including secondary osteoporosis [47, 48].

We acknowledge our study has some limitations. Firstly, the sample size is small and might reduce statistical power. Secondly, the absence of longitudinal data on controls does not allow for an adequate comparison between the two groups.

In conclusion, our study shows that footballers have significantly higher serum irisin values than the general population. Further studies are necessary with a larger population and a prolonged period of observation to completely understand the role of this myokine in the cross-talk between muscle and bone.
